# Natural anticancer agents: prospection of medicinal and aromatic plants in modern chemoprevention and chemotherapy

**DOI:** 10.1007/s13659-025-00511-0

**Published:** 2025-04-21

**Authors:** Patricia Quintero-Rincón, Karina Caballero-Gallardo, Jesus Olivero-Verbel

**Affiliations:** 1 Research Group Design and Formulation of Medicines, Cosmetics, and Related, Faculty of Pharmaceutical and Food Sciences, Universidad de Antioquia, 050010 Medellín, Colombia; 2 Functional Toxicology Group. School of Pharmaceutical Sciences, Zaragocilla Campus, Universidad de Cartagena, 130014 Cartagena, Colombia; 3 Environmental and Computational Chemistry Group, School of Pharmaceutical Sciences, Zaragocilla Campus, Universidad de Cartagena, 130014 Cartagena, Colombia

**Keywords:** Medicinal and aromatic plants, Chemoprevention, Chemotherapy, Vegetable extracts, Essential oils, Natural volatiles, Cancer cell lines

## Abstract

Natural products obtained from medicinal and aromatic plants are increasingly recognized as promising anticancer agents due to their structural richness, including terpene and flavonoid molecules, which induce apoptosis and modulate gene expression. These compounds offer an alternative to conventional treatments, often costly, which face challenges such as multidrug resistance. This review aims to provide a promising alternative approach to effectively control cancer by consolidating significant findings in identifying natural products and anticancer agent development from medicinal and aromatic plants. It synthesizes the findings of a comprehensive search of academic databases, such as PubMed and Springer, prioritizing articles published in recognized peer-reviewed journals that address the bioprospecting of medicinal and aromatic plants as anticancer agents. The review addresses the anticancer activities of plant extracts and essential oils, which were selected for their relevance to chemoprevention and chemotherapy. Compounds successfully used in cancer therapy include Docetaxel (an antimitotic agent), Etoposide VP-16 (an antimitotic agent and topoisomerase II inhibitor), Topotecan (a topoisomerase I inhibitor), Thymoquinone (a Reactive Oxygen Species-ROS inducer), and Phenethyl isothiocyanate (with multiple mechanisms). The review highlights natural products such as Hinokitiol, Mahanine, Hesperetin, Borneol, Carvacrol, Eugenol, Epigallocatechin gallate, and Capsaicin for their demonstrated efficacy against multiple cancer types, including breast, cervical, gastric, colorectal, pancreatic, lung, prostate, and skin cancer. Finally, it highlights the need for continued bioprospecting studies to identify novel natural products that can be successfully used in modern chemoprevention and chemotherapy.

**Graphical Abstract:**

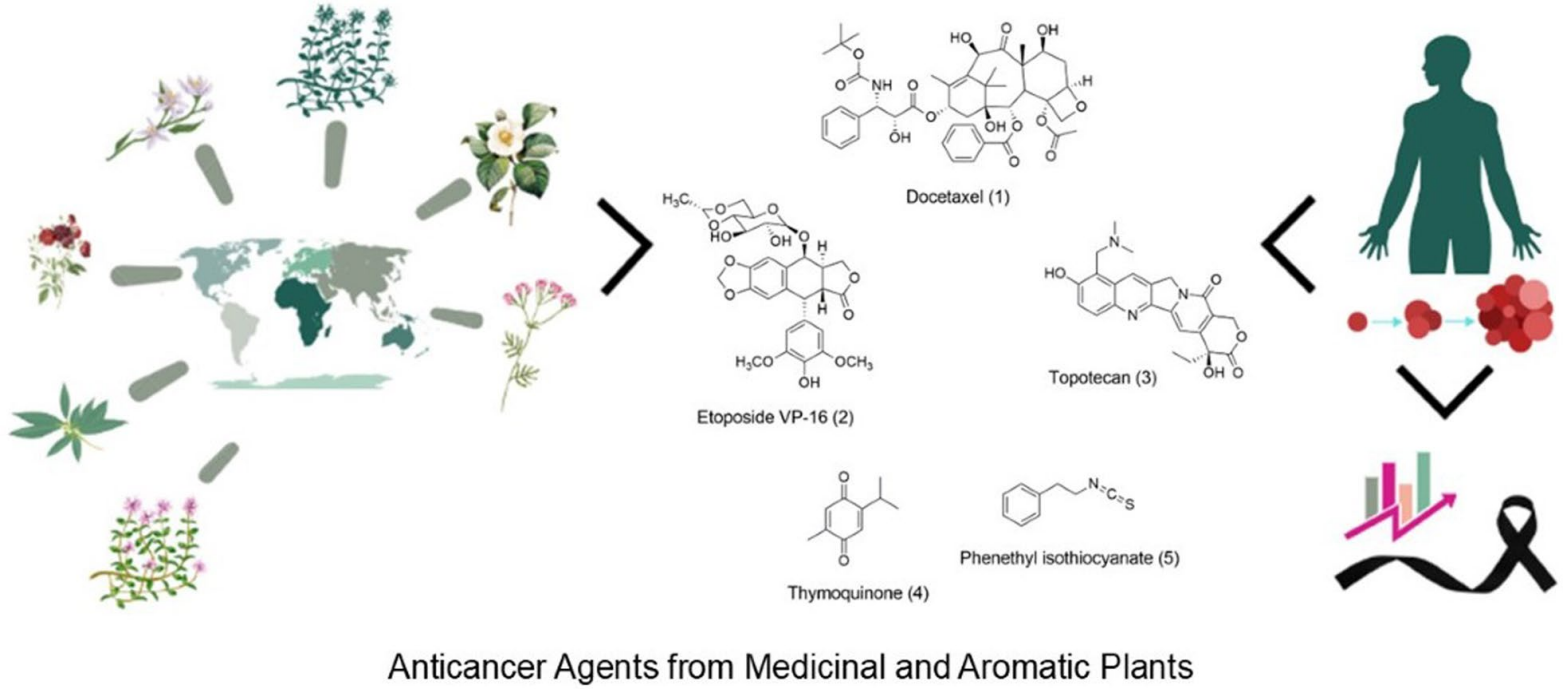

## Introduction

Cancer is a term that encompasses a diverse group of diseases characterized by the uncontrolled proliferation of abnormal cells, which can affect humans in any part of the body during their life cycle [1]. Under normal conditions, human cells form and multiply to replace cells that age or become damaged; in this sense, aging and cellular dynamics, including cell death and the division of young cells, are crucial for maintaining healthy living. Disruption of this balance can lead to aberrant cell proliferation, resulting in tumors that invade surrounding tissues and spread through the bloodstream and lymphatic system [2]. This process, known as metastasis, is a major cause of cancer-related deaths [1, 2]. The disease is marked by the abnormal expression of the phosphatidylinositol 3-kinase (PI3K) pathway [3], leading to sustained cell proliferation, and the loss of tumor-suppressive functions of the p53 [4], and retinoblastoma protein (pRB) pathways [5].

Despite advancements in anticancer strategies such as surgery, radiotherapy, chemotherapy, and immunotherapy, challenges persist, including immune system suppression, high treatment costs, and drug resistance in tumor cell lines [5, 6]. The significant mortality associated with conventional treatments underscores the need for innovative therapeutic approaches [7].

According to statistics from the World Health Organization (WHO) and the International Agency for Research on Cancer (IARC), cancer represents a significant public health challenge, being the second leading cause of death worldwide [8]. An analysis by Rajappa et al. [9] highlights the uneven distribution of cancer incidence and mortality, evidencing a clear correlation between socioeconomic development and cancer rates in Asian countries, where access to effective anticancer treatments is severely limited due to their high costs.

The most recent update from the Global Cancer Observatory (GCO) (https://gco.iarc.fr/ accessed January 5, 2025) places Latin America and the Caribbean (LAC) in fourth place with 7.8% of new cancer cases in 2022, after Asia (49.2%), Europe (22.4%), and North America (13.4%). According to estimates by the Pan American Health Organization (PAHO)/WHO (https://www.paho.org/es/ accessed January 5, 2025), 40% of cancer cases could be prevented by reducing key risk factors. These risk factors are attributable to infections by viruses: *Human papillomavirus* (HPV), Hepatitis B virus (HBV), and Hepatitis C virus (HCV); bacteria (*Helicobacter pylori*), and other microbial agents. Other risk factors are associated with the consumption of alcoholic beverages by the amount of alcohol consumed (moderate, < 20 g/day; risky, 20–60 g/day; heavy, > 60 g/day), obesity (according to anatomical site: breasts, corpus uteri, colon, kidney, gallbladder, pancreas, rectum, oesophageal adenocarcinoma, and ovary), and exposure to ultraviolet radiation.

The strategies proposed by PAHO/WHO include improving access to affordable chemotherapeutic drugs for the population and promoting palliative care policies, as programs for managing symptoms (https://www.paho.org/ accessed January 5, 2025). In response to this global health demand, the study of new natural alternatives for cancer chemoprevention or chemotherapy and its risk factors are linked to PAHO strategies, where scientific knowledge of medicinal and aromatic plants plays a fundamental role in mitigating the damage caused by this disease [10].

Cultural transmission of ethnobotanical knowledge has been globally essential to folk medicine practices [11]. For millennia, medicinal and aromatic herbs have played a crucial role in health rituals conducted by shamans and sorcerers, who incorporated these plants into potions, antidotes, ointments, and cosmetics. Today, these practices form the foundation of traditional medicine in many cultures [12, 13]. This accumulated knowledge, honed through trial and error, continues to thrive, particularly in regions conducive to cultivating these species [14, 15].

Historically, ancient Near Eastern civilizations introduced the traditional use of medicinal and aromatic plants as anticancer agents. Persian, Mesopotamian, Greek, and Roman civilizations greatly influenced these medicinal practices, followed later by influences from Arab and Islamic civilizations [16]. This knowledge subsequently spread globally, and in America, it occurred especially during colonization, where a dynamic and active process of cultural exchange between different groups of people was generated through various types of migratory events [17].

The literature review highlights in vitro studies of the cytotoxic and antiproliferative potential of natural products obtained from plants against cancer cell lines. Several herbs from different origins have been studied for their demonstrated bioactivities in traditional practice. Results of in vivo trials and clinical studies in cancer patients using whole plants, phytochemicals, or their extracts have shown that these exert chemopreventive or chemotherapeutic effects and reduce adverse events of anticancer drugs and disorders associated with this disease [18].

Essential oils (EOs) are noteworthy in anticancer strategy studies due to their chemical nature. The variety of volatile constituents involves different mechanisms to exert biological action, including DNA repair, cell cycle arrest, apoptosis, inhibition of metastasis, and multidrug resistance. This latter is the greatest challenge in cancer treatment [10]. However, medicinal and aromatic plant extracts have shown promising results, especially for obtaining isolated compounds due to their diversity, high availability, and low toxicity in cells in healthy conditions [14].

This review underscores the importance of traditional knowledge and continued research into natural products to identify novel therapeutic agents for cancer prevention and treatment. It aims to highlight the potential of medicinal and aromatic plants in developing innovative anticancer drugs. It focuses on the anticancer properties of chemical compounds, including plant extracts and EOs, which have been evaluated against various cancer cell lines.

## Methodology

Information was collected through academic search systems for articles published in peer-reviewed journals, including PubMed, Springer, Science Direct, Scopus, Google Scholar, and ResearchGate. The search terms used were "medicinal and aromatic plants", "essential oils", "anticancer", "chemoprevention", "chemotherapy", "phytocompounds", "volatile components" or "natural volatiles" or "cancer cell lines".

### Inclusion criteria

- Priority was given to articles published in recognized peer-reviewed journals that address the topic of Bioprospecting of medicinal and aromatic plants. Additionally, updates in the area of ​​interest published by GCO and PAHO/WHO were included.
- Studies that directly addressed the anticancer activities of plant extracts and essential oils and their applications in chemoprevention and chemotherapy were selected.
- Recent publications were prioritized. However, classical references were also included.

### Exclusion criteria

- Studies that were not directly related to the anticancer properties of plants or topics not addressed in the review were omitted.
- Duplicate publications were excluded.

## Harnessing nature's defenses: the role of natural products in anticancer drug discovery

During the search for new drugs with anticancer potential, a chemical screening of the extracts and biological activity assays are necessary for rapid progress in obtaining new molecules. Screening of selected plant extracts by their use in traditional medicine can lead to potential anticancer agents to block, retard, or reverse the carcinogenic [19]. However, field observations are pivotal. In this regard, Hostettmann et al. [20] indicated that: “*A plant species growing in a hostile environment, such as warm and humid tropical forests, will attempt to protect itself by synthesizing insecticidal, fungicidal, antibacterial, or virucidal constituents. Then, for example, if the leaves of a plant show no signs of aggression, they may contain defensive compounds against insects or microorganisms*”. That is to say, the plants adjust their physiological states in response to a stressful environment (stress both biotic and abiotic, climate changes, and phenological growth phases, among others) to improve their well-being and survival. These adjustments involve modulation of secondary metabolite production pivotal for the species' survival, mainly antioxidants, including terpenoids, alkaloids, and phenolics [21].

It has been estimated that currently two-thirds of anticancer drugs are obtained from plant extracts, and classification according to the pharmacological effects places them as antimitotics, topoisomerase inhibitors (Topo I and Topo II), ROS inducers, angiogenesis inhibitors, and histone deacetylases (HDAC) inhibitors [22]. In this sense, antimitotic drugs induce cell cycle arrest and tumor cell death [23], topoisomerase inhibitors act via topoisomerase poisoning leading to replication fork arrest and double-strand break formation [24], ROS inducers cause oxidative stress-induced apoptosis in cancer cells [25], angiogenesis inhibitors act on an endothelial cell in the growing vasculature (direct inhibitors) or block the activity of angiogenesis inducers (indirect inhibitors) [26], and HDAC inhibitors (epigenetic therapy) induce cell death in a select subpopulation of cells, restricted to the treatment of hematological malignancies [27, 28].

**Fig. 1:**
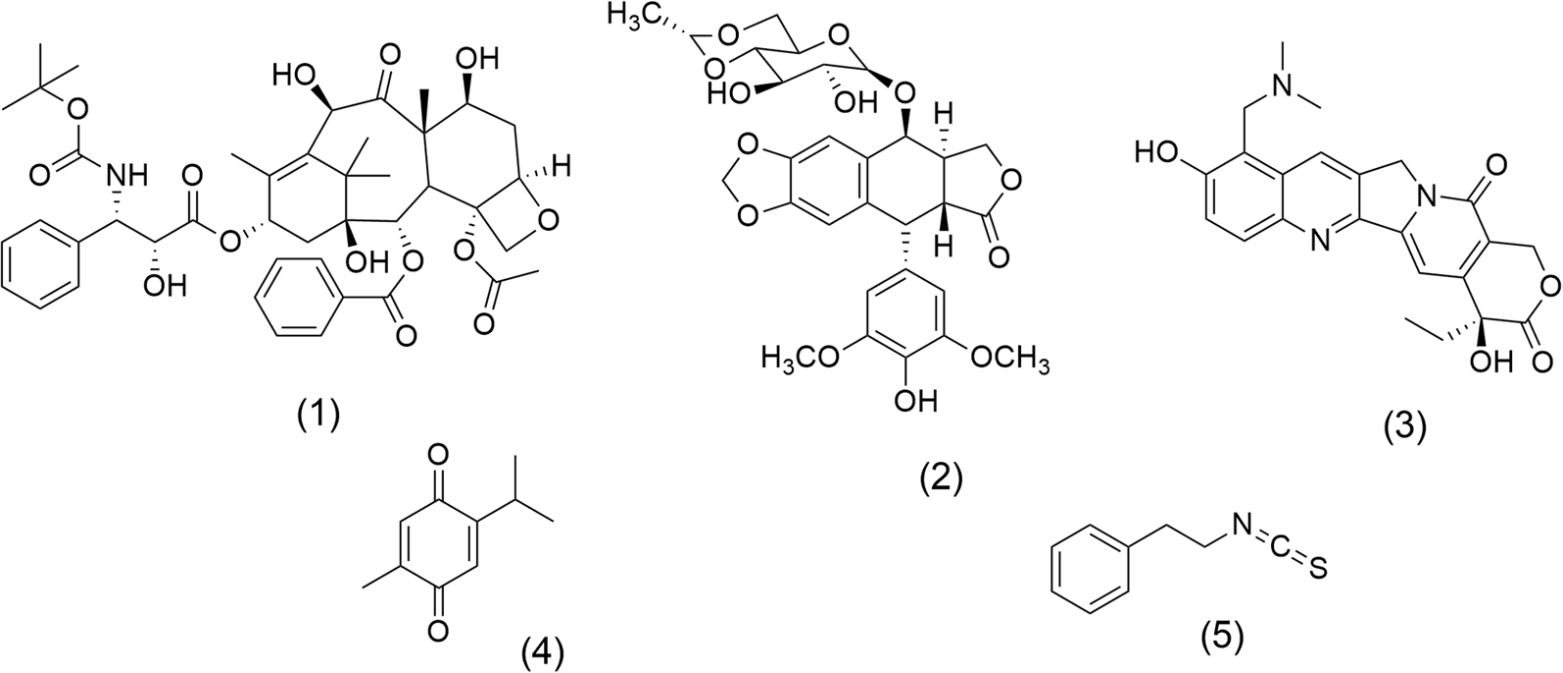
Chemical structures of recognized anticancer agents: Docetaxel (**1**), Etoposide VP-16 (2), Topotecan (3), Thymoquinone (4), and Phenethyl isothiocyanate (5)

Figure 1 shows the chemical structures of some anticancer drugs derived from secondary metabolites in plant extracts. Docetaxel (1), an antimitotic agent obtained by hemisynthesis of baccatin III, extracted from *Taxus* spp. (Taxaceae family), and a structural analogue of Paclitaxel (Taxol), is used in castration-resistant prostate cancer [29]. Another antimitotic agent and Topo II inhibitor recognized is Etoposide, VP-16 (2), obtained from *Podophyllum peltatum* L. (Berberidaceae family) [30], which is used to manage various types of cancer, including lung, bladder, stomach, testicular, and prostate [31]. Topotecan (3) isolated from *Camptotheca acuminata* Decne. from Nyssaceae family acts as a potent Topo I inhibitor agent [32], Thymoquinone (4) is a ROS inducer obtained from *Nigella sativa* L. (Ranunculaceae family) [33], and Phenethyl isothiocyanate (5), an anticancer agent whose activity is mediated by various mechanisms, including histone deacetylase inhibitor [34], induction of apoptosis, inhibition of cell proliferation, suppression of angiogenesis, and reduction of metastasis [35]. The latter is isolated from *Nasturtium officinale* L. (Brassicaceae family) [34].

Today, the antioxidant molecules from nature, including natural products obtained from plants, are remarkable due to their anticancer potential. Some belong to terpenes, alkaloids, flavonoids, tannins, and phenolic groups [36]. The US Food and Drug Administration (US FDA) has approved several of these molecules; others are in the experimental phase. Moreover, some plant species contain bioactive molecules that provide therapeutic benefits synergistically [37].

**Fig. 2:**
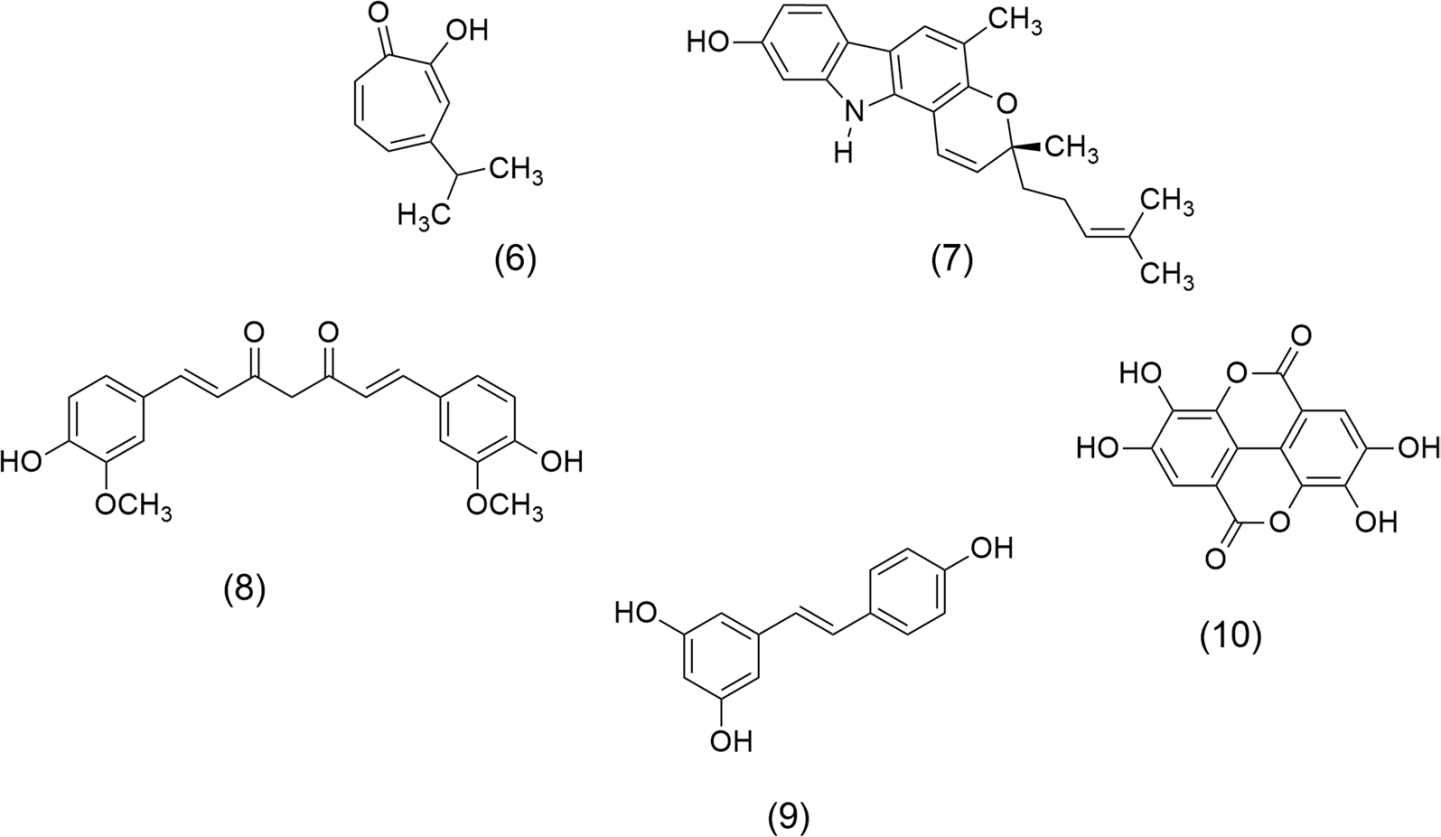
Chemical structures of terpene, alkaloid, and polyphenolic with anticancer properties: Hinokitiol (6), Mahanine (7), Curcumin (8), Resveratrol (9), and Ellagic acid (10)

Literature underscores terpenes and alkaloids as key molecules against cancer. Hinokitiol (β-thujaplicin) (6), and Mahanine (7) (Fig. 2) are diterpene and alkaloid, respectively, isolated from *Chamaecyparis obtusa* (Siebold & Zucc.) Endl. (Cupressaceae) and *Murraya koenigii* (L.) Spreng. Rutaceae (also called curry tree), have highlighted the importance of plants as a source of biologically active molecules against cancer. Investigations have shown that (6), a natural bioactive monoterpenoid, exerts a potential anticancer effect on metastatic melanoma cells (B16-F10) due to their cell inhibition through downregulation of survivin protein, which activates the extracellular signal-regulated kinases (ERK)/mitogen-activated protein kinase phosphatase-3 (MKP-3)/proteosome pathway [38]. Furthermore, this monoterpene inhibits the heparanase via extracellular signal-regulated kinase and protein kinase B pathway leading to the reduction of tumor metastasis [39]. Recently, Chen et al. [40] showed that (6) has antitumor properties on endometrial cancer since it induces apoptosis mediated by ROS and p53-driven cell-cycle arrest in cell lines of Ishikawa and human endometrial adenocarcinoma (HEC-1A). On the other hand, (7) is a carbazole alkaloid that interferes with tumor growth and metastasis development by stimulating cell cycle arrest and decreasing apoptotic and anti-apoptotic proteins [41]. This compound inhibits the viability of estrogen receptor-positive breast cancer (ER+)/ wild type of p53 (p53WT) MCF-7 and triple-negative/p53Mut MDA-MB-231 cells, by apoptosis and arresting the cells in G0/G1. Also, (7) showed a significant reduction of mammary tumor burden induced by *N*-Methyl-*N*-nitrosourea (MNU) in a rat model [42]. Furthermore, (7) disrupts cell migration, invasion, and PI3K/protein kinase B (AKT)/mechanistic target of rapamycin (mTOR) signaling pathway in human glioma cell line (HS-683) and inhibits tumor growth in vivo [43].

Polyphenols such as Curcumin (8) and Resveratrol (9), shown in Fig. 2, are promising anticancer molecules. Curcumin (8) is obtained from *Curcuma longa* L. (Zingiberaceae). The anticancer activity of (8) is due to antiproliferative and apoptotic effects by modulation of multiple molecular targets, among them, transcription factors (activator protein-1, AP-1; early growth response factor-1, EGR-1; signal transducer and activator of transcription-3 STAT-3; nuclear factor kappa B, NF-кB), cytokines (interleukin IL-2, IL-6, IL-8, macrophage inflammatory proteins MIP, tumor necrosis factor alpha TNF-α), receptors (estrogen receptor alpha, Erα; human epidermal growth factor receptor-2, HER-2; Fas receptor, Fas-R; epidermal growth factor receptor, EGFR), enzymes (enzyme that hydrolyses adenosine triphosphate, ATPase; Glutathione S-transferase, GST; cyclooxygenase 2, COX-2; telomerase), growth factors (epidermal growth factor, EGF; platelet-derived growth factor, PDGF; hepatocyte growth factor, HGF), and kinases (mitogen-activated protein kinase, MAP-K; protein kinase A, PKA; protein tyrosine kinase, PTK; protein kinase B, PKB; p21-activated kinase, PAK) [44]. Resveratrol (9), a stilbenoid isolated from *Polygonum cuspidatum* Sieb. et Zucc. (Polygonaceae), affects signal-transduction pathways that control cell growth and its division, metastasis, apoptosis, angiogenesis, and inflammation [45], modulates cell signaling molecules, among them cytokines, caspases, matrix metalloproteinases (MMPs), and nuclear factor kappa B (NFκB) [46]. Ellagic acid (10) is another important polyphenol extracted from *Punica granatum* L. (Punicaceae), which acts by inhibiting tumor-cell migration, invasion through the extracellular matrix, and angiogenesis. It has shown anticancer activity against colorectal, breast, prostate, lung, bladder, and ovarian cell lines [47].

**Fig. 3:**
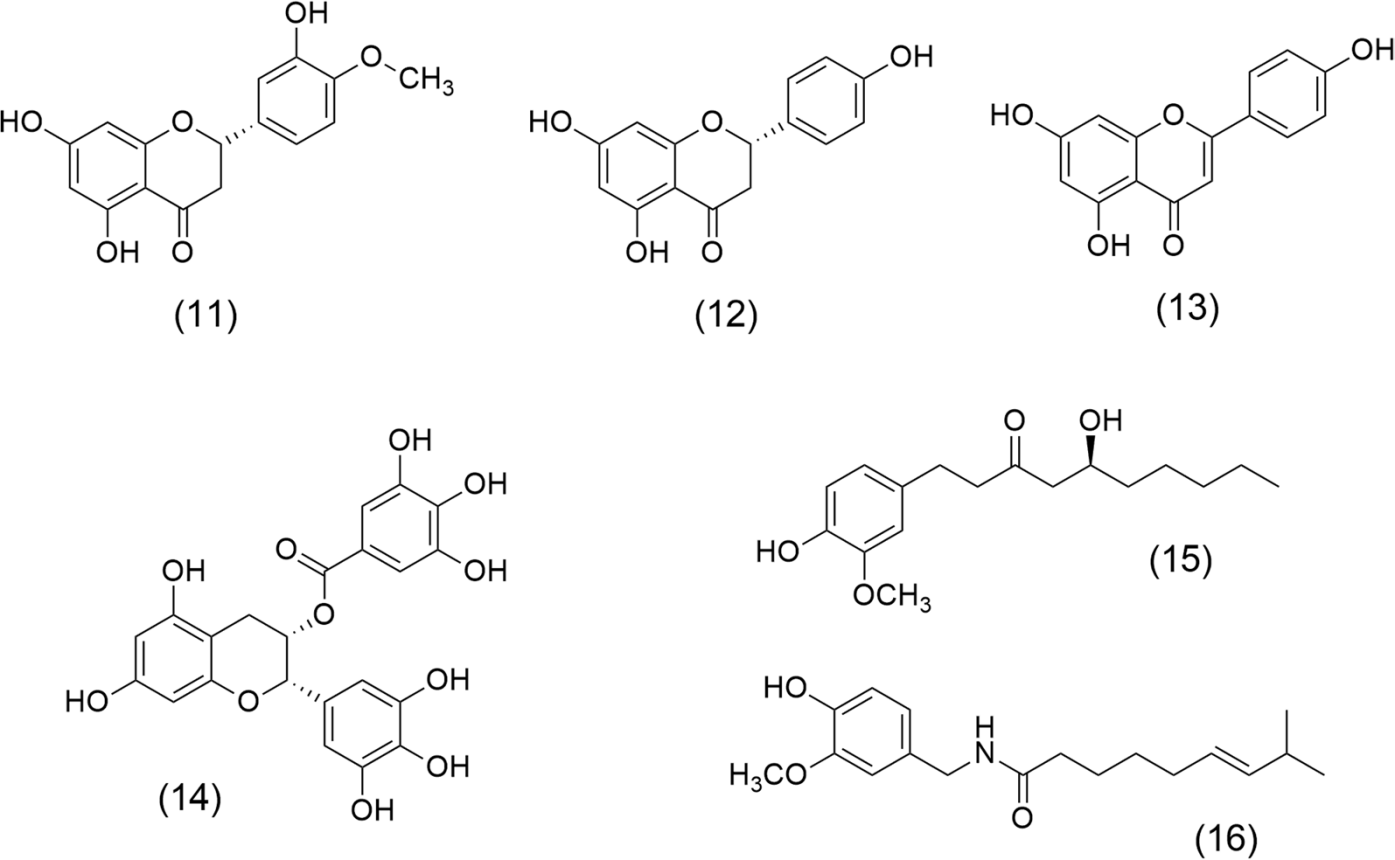
Chemical structures of flavonoid, tannin, and phenolic molecules with anticancer properties: Hesperetin (11), Naringenin (12), Apigenin (13), Epigallocatechin gallate (14), Gingerol (15), and Capsaicin (16)

On the other hand, flavonoids are the most abundant polyphenols isolated from aromatic and medicinal plant extracts; these molecules have therapeutic effects in preventing cancer and related chronic diseases. Therefore, they are used by their health-promoting properties as nutraceuticals [48]. Hesperetin (11), Naringenin (12), and Apigenin (13) (Fig. 3) are three flavonoids with anticancer potential. (11) and (12), are found in *Origanum acutidens* (Hand.-Mazz.) Ietsw. (Lamiaceae) and in citrus fruits, including lemon, and orange. These are two flavonoids with chemotherapeutic and chemosensitising activities. (11), a dihydroflavone, induces G2/M phase cell-cycle arrest and apoptosis in human glioblastoma cells by p38 MAP-K activation [49]. (12) inhibits tumor cell proliferation and angiogenesis in malignant melanoma as B16F10 murine and SK-MEL-28 cells [50]. Combined treatment with (11) and (12) inhibits the proliferation of pancreatic cancer (Miapaca-2 and Panc-1 cells) by induction of caspase-3 cleavage, also acts by inhibition of migration of human pancreatic cancer cells, the phosphorylation of focal adhesion kinase (FAK), and p38 signaling [51]. (13) is a flavone from *Origanum vulgare* L. (Lamiaceae). This flavone induces apoptosis, autophagy, and immune response. Also, it inhibits cell cycle progress, cell migration, and invasion by modulating the targeting of multiple signaling pathways. Its beneficial effects include colorectal, breast, lung, prostate, ovarian, pancreatic, and cervical cancer, renal cell, adenoid cystic, thyroid, head and neck squamous cell carcinoma, and oral squamous cell. Furthermore, (13) inhibits melanoma growth, leukemia, glioblastoma, mesothelioma, and osteosarcoma [52].

Epigallocatechin gallate (14) obtained from *Camellia sinensis* (L.) O. Kuntze. (Theaceae) is the most representative molecule of the tannins; it is an epigallocatechin and gallic acid ester, which induces apoptosis and cell cycle arrest. It has anti-inflammatory effects and modulates epigenetic changes in gene expression and chromatin organization via interaction with deoxyribonucleic acid (DNA) methyltransferase and histone deacetylases (HDACs) [53]. (14) has shown satisfactory results in studies of breast, lung, colorectal cancer, osteosarcomas, and neuroblastoma [54]. Gingerol (15) is an important phenolic compound that modulates NFκB, signal transducer and activator of transcription 3 (STAT3), AP-1, EGFR, vascular endothelial growth factor receptor (VEGFR), MAP-K, TNF-α, and COX-2 [55]. (15) obtained from *Zingiber officinale* Roscoe (Zingiberaceae), inhibits the proliferation of breast cancer cells (MCF7), suppresses oral cancer cell growth via activation of AMP-activated protein kinase (AMPK), and suppresses the AKT/mTOR signaling pathway [7, 56]. Finally, Capsaicin (16) obtained from *Capsicum annuum* L., Solanaceae is a phenolic compound that acts as a carcinogen or cancer chemoprevention agent. It treats lung, breast, colorectal, stomach, prostate, pancreatic, bladder cancer, nasopharyngeal carcinoma, cholangiocarcinoma, osteosarcoma, melanoma, fibrosarcoma, and glioblastoma [57].

**Fig. 4:**
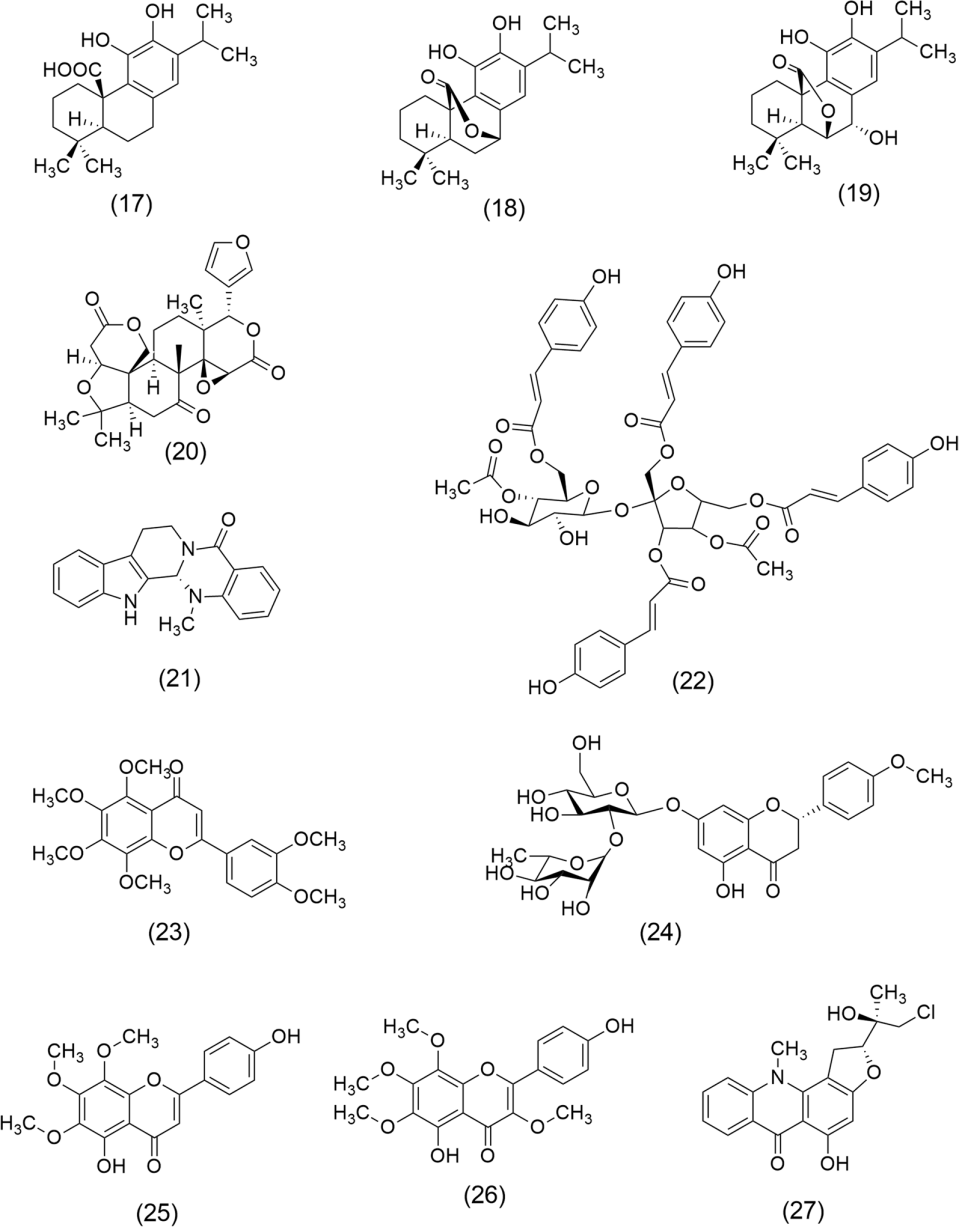
Chemical structures of Carnosic acid (17), Carnosol (18), Rosmanol (19), Limonin (20), Evodiamine (21), Polygonumins A (22), Nobiletin (23), Poncirin (24), Xanthomicrol (25), Calycopterin (26), and Isogravacridonechlorine (27)

Currently, computational tools are used with a biological indicative parameter, e.g., methyl thiazolyl tetrazolium (MTT) assay-based cell lines studies [58], which has allowed rapid progress in identifying and obtaining new anticancer molecules. Numerous studies of plant extracts have been evaluated for their cytotoxic, antiproliferative, antiangiogenic, and apoptotic properties. Some of the most significant findings of plant extracts and their phytocompounds (Fig. 4) are those described below.

*Rosmarinus officinalis* L. extract (known as rosemary) is a potent antioxidant agent and anti-inflammatory, which has been reported for anticancer properties attributed to diterpenes, including Carnosic acid (17), Carnosol (18), and Rosmanol (19), that act on key signaling pathways [59]. This aromatic plant belongs to the family Lamiaceae and is native to the Mediterranean region. Traditionally, rosemary has been recognized for its culinary uses and medicinal properties [60]. European Union (EU) approved its use as extracts standardized to diterpenes, e.g., (17) and (18). Also, the US FDA granted it the status of Generally Recognized as Safe (GRAS) [61]. Anticancer activities reported include colon cancer (extract showed strong inhibition of proliferation, migration, and colony formation of colon cancer cells regardless of their phenotype) [62], hepatocellular carcinoma (inhibited proliferation of HepG2 Cells) [63], lung cancer (extract decreased the activation of AKT/mTOR/p70S6 kinase (p70S6K) and showed inhibited proliferation and survival of A549 cells) [64], skin cancer (anti-proliferative effect on Human Melanoma A375 Cells) [65], oral cancer (rosemary exerts anti-inflammatory effects, proapoptotic, antiproliferative, and anti-angiogenic potential in buccal pouch carcinogenesis in a hamster model) [66], prostate cancer (extract inhibits prostate cancer cell proliferation and survival by targeting AKT and mTOR) [67], and breast cancer (extract exerts antiproliferative effects, inhibits survival, AKT, and mTOR signaling in triple-negative breast cancer cells) [68].

*Evodia rutaecarpa* (Juss.) Benth (Rutaceae) also called Euodia or Bee bee tree in the west, is a rich source of anticancer molecules, among them Limonin (20) and Evodiamine (21), which have been studied by their effects on ovarian cancer cells and colon cancer cells, respectively. Euodia extract and (20) showed a significant reduction in ovarian cancer cell lines SKOV-3, A2780, and RMUG-S by inducing apoptosis via activation of the p53 signaling pathway [69]. Furthermore, (21) a quinolone alkaloid exhibited prominent anti-proliferation and apoptosis-inducing effects in HCT-116 cells (colon cancer), mediated by bone morphogenetic protein 9 (BMP9) upregulation, which can activate p53 through upregulation of hypoxia-inducible factor 1-alpha (HIF 1α) [70].

*Polygonum minus* Huds (Polygonaceae), or kesum, is an aromatic plant that grows in temperate regions in Southeast Asian countries, it is used as a spice in the food industry. Methanol extract of kesum contains Polygonumins A (22), a potential anticancer agent whose structure includes four phenylpropanoid ester units and a sucrose unit, was evaluated on human cancer cell lines, including leukemia cells (K562), breast adenocarcinoma cells (MCF7), and colorectal cancer cells (HCT116) [71].

*Citrus reticulate* L. (mandarin orange) belongs to the Rutaceae family and is native to Southeast Asia and the Philippines. Mandarin orange peel was evaluated on the DLA cell line (Dalton´s Lymphoma Ascites) showing inducing of apoptosis by G0/G1 phase cell cycle arrest in DLA cells, as well as nuclear condensation, membrane blebbing, formation of apoptotic bodies, and DNA damage. These anticancer effects were attributed to polymethoxy flavones in water extract, e.g., Nobiletin (23) [72]. Furthermore, (23) was reported by suppressing autophagic degradation by over-expressing the AKT pathway, also inducing apoptosis in multidrug-resistant SKOV3/TAX ovarian cancer cells [73]. Another compound flavanone glycoside isolated from mandarin orange is Poncirin (24), which exerts antiproliferative effects on SGC-7901 gastric cancer cells [74].

*Dracocephalum kotschyi* Boiss (Lamiaceae), is an endemic species of Iran commonly known as Badrandjboie-Dennaie. This plant has been amply used as an anticancer agent, especially in leukemia treatment. Xanthomicrol (25) and Calycopterin (26) are two trimethoxylated hydroxyflavones derived from the leaves of *D. kotschyi*. These compounds are noted for their immunoinhibitory properties, contributing to their potential therapeutic applications. Studies have demonstrated that (26) exerts antiproliferative effects on HepG2 cells by inhibiting cell cycle progression at the G2/M transition, which leads to growth arrest and apoptosis, increases intracellular levels of ROS, NO, and decreases the expression of mitotic kinase cdc2, mitotic phosphatase cdc25c, mitotic cyclin B1, and apoptotic factors pro-caspases-3 and -9 [75]. On the other hand, (25) inhibits both B16F10 melanoma cell viability and cancer cell growth in an in vivo model by angiogenesis inhibition [76].

*Ruta graveolens* L. (Rutaceae), popularly called rue, is a species native to southern Europe, recognized for its culinary and medicinal uses. Roots and aerial parts extract of rue contains alkaloids, including Isogravacridonechlorine (27), which exerts a marked effect on the viability of MDA-MB-231 breast cancer cells. (27) disturbs the cell cycle by decreasing the G2/M and G0/G1 and increasing the S phase and the appearance of the subdiploid (sub-G1) population. Furthermore, it activates caspase-3 and -9, but not caspase-8, indicating an activation of an intrinsic apoptotic pathway in MDA-MB-231 cells [77].

**Fig. 5:**
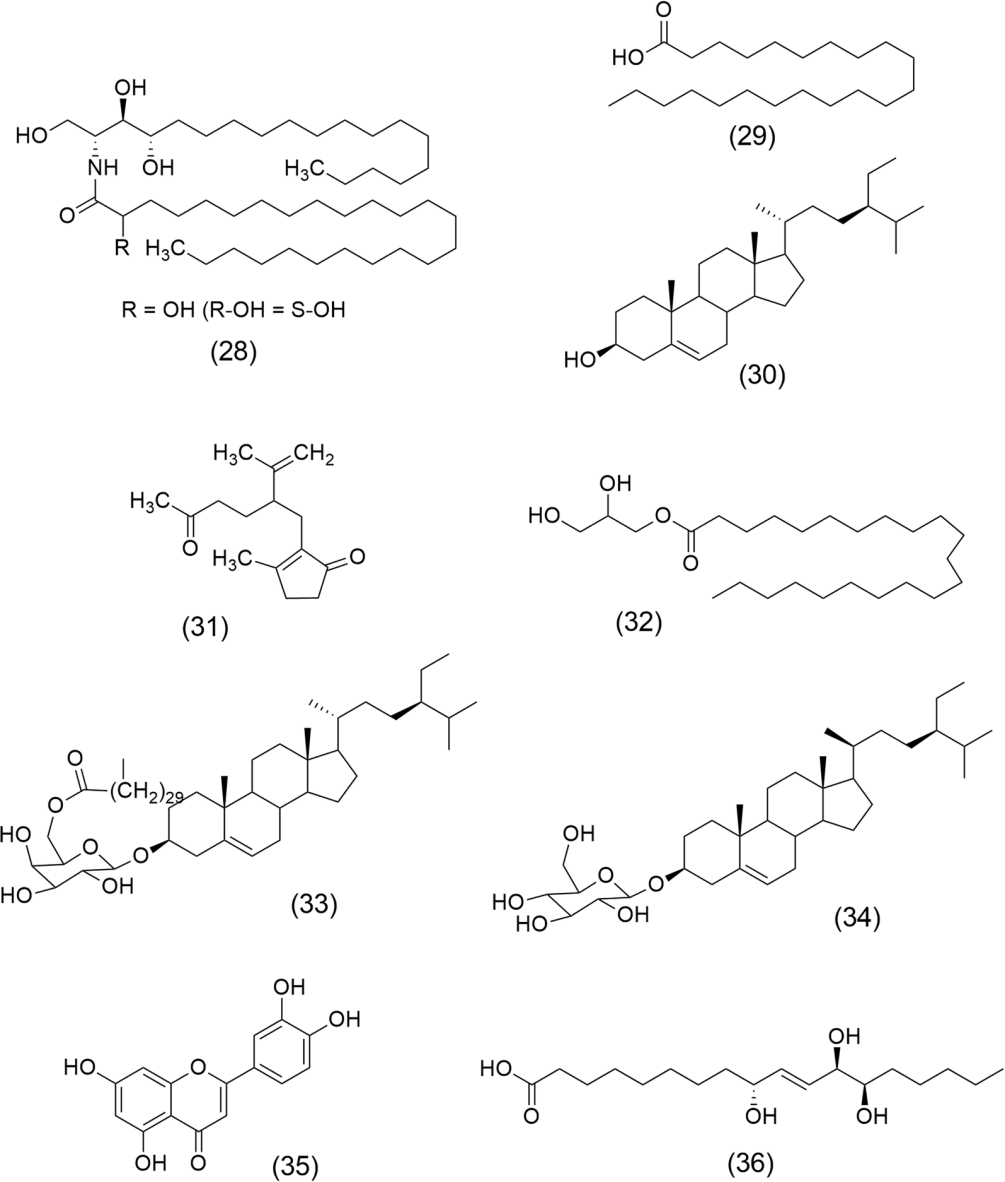
Chemical structures of anticancer constituents from *Cyperus rotundus* L.: 2′S-[2-Hydroxypentacosanoylamino]-1′,3′S,4′R nonadecanetriol (28), Behenic acid (29), β-Sitosterol (30), Mandassidione (31), Behenic acid monoglyceride (32), Sitosteryl (6`-hentriacontanoyl)-β-D-galactopyranoside (33), β-Sitosterol 3-O-β-D-glucoside (34), Luteolin (35), and Pinellic acid (36)

*Cyperus rotundus* L. (Cyperaceae) also known as purple nutsedge is a perennial plant widely used in Traditional Chinese Medicine. Studies have shown anticancer effects on human triple-negative breast cancer (TNBC) cells [78]. Samra et al. [79] showed the anticancer activity of the methanolic extract of *C. rotundus* and compounds isolated from subfractions: 2′S-[2-Hydroxypentacosanoylamino]-1′,3′S,4′R nonadecanetriol (28), Behenic acid (29), β-Sitosterol (30), Mandassidione (31), Behenic acid monoglyceride (32), Sitosteryl (6`-hentriacontanoyl)-β-D-galactopyranoside (33), β-Sitosterol 3-O-β-D-glucoside (34), Luteolin (35), and Pinellic acid (36) (Fig. 5). Cytotoxicity of these phytocompounds was demonstrated against human hepatocellular carcinoma (HepG2), prostatic adenocarcinoma (PC3), and breast cancer (MCF-7) cell lines using the MTT assay.

Corsican-Sardinian (*Santolina corsica* Jord. & Fourr., Asteraceae), found only in Monte Albo (Sardinia), exhibits anticancer potential on MDA-MB-231 cells using n-hexane and methanolic extracts. Bonesi et al. [80] showed that both n-hexane and methanolic extracts, trigger apoptosis, and reduce invasive and migratory capacities of MDA-MB-231 cells.

*Chenopodium album* L. (Chenopodiaceae), commonly known as lamb's quarters, melde, goosefoot, wild spinach, fat-hen, cinzo, or quinhuilla, is amply used in traditional Chinese medicine. Its petroleum ether extract exhibited growth inhibitory effects (dose- and time-dependent) and G1 phase cell cycle arrest, as well as cell apoptosis (exhibited dose-dependent) on A549 human non-small cell lung cancer [81].

*Petiveria alliacea* L. (Phytolaccaceae) or “anamu” is a perennial herb, widely used by traditional medicine in the Caribbean, and in Central and South America [82]. Leaves and stem extracts of anamu showed anticancer effects in a murine breast cancer model using 4T1 cells. A fraction of the extract affects the glycolytic pathway enzymes and induces cell death and tumor regression in vitro and in vivo models, respectively [83].

*Angelica archangelica* L. (Apiaceae) or holy ghost root is an aromatic plant used in traditional medicine for its beneficial properties. Oliveira et al. [84] showed that crude extract of holy ghost root was cytotoxic against breast adenocarcinoma cells, especially on 4T1 and MCF-7, but not for human fibroblasts.

Cinnamon (*Cinnamomum cassia* Presl, Lauraceae), or the eternal tree of tropical medicine, is one of the important spices for health enhancement worldwide [85]. In a study, ethyl acetate, chloroform, and hexane extracts of *C. cassia* exhibit antiproliferative effects and DNA damage on breast cancer cells (MCF-7) [86]. Park et al. [87] showed cytotoxic activity in human colorectal cancer cells through the suppression of cell proliferation and the induction of apoptosis. Further, apoptotic induction but not autophagic cell death by *C. cassia* extract was evaluated on human oral cancer cells [88].

In *Cymbopogon citratus* (DC.) Stapf, a member of the Poaceae family, has several polysaccharides identified in its aqueous extract, which exhibits anticancer activity against human cervical cancer (Siha) and prostate cancer (LNCap) cell lines. The extract activates the intrinsic apoptotic signaling pathway, through the upregulation of caspase 3 and downregulation of Bcl-2 family genes, leading to the release of cytochrome C [89, 90]. Additionally, the essential oil derived from this plant has been shown to induce cell cycle arrest and apoptosis in A549 human lung cancer cells [91].

Recent studies have shown that *Dioon rzedowskii* (Zamiaceae family) effectively inhibits cell growth in MCF-7 and HepG2 cell lines by suppressing cell proliferation and the induction of apoptosis. The observed anticancer effects were linked to an increased expression of p53 and Bax and a reduction in cyclin D1 levels, which was associated with lower levels of phosphorylated MAPK kinases [92].

*Pongamia pinnata* Linn, a member of the Fabaceae family, is recognized for its significant role in herb-based traditional medicine. This study investigates the anticancer and antioxidant properties of its leaf extract. The phytochemical analysis was followed by in-silico assessments of anticancer receptors with ligands through molecular docking and simulation. The study revealed incremented phenolic and flavonoid content and anticancer potential against A431 skin cancer cells, suggesting that *P. pinnata* is a valuable source for drug development targeting melanomas [93].

Saffron (*Crocus sativus* L.), a traditional medicinal herb of the Iridaceae family, is rich in active compounds known for their anticancer properties. Enrichment analyses indicated that saffron enhances Th17 cell differentiation and IL-17 signaling pathway, effectively suppressing the proliferation of CT26 and HCT116 cells. These findings suggest that the active components of saffron improve the immune microenvironment of tumors, increasing the efficacy of immunotherapy for colorectal cancer [94].

The anticancer activity of an ethanolic extract of leaves from *Myrtus communis* Linn. (Myrtaceae family) was evaluated against various cancer cell lines, including breast (MCF-7), liver (HepG2), cervical (HeLa), and colon (HCT116) cancers. The extract demonstrated potent cytotoxic effects, primarily through apoptosis and cell cycle arrest in the G1 phase, suggesting a significant potential for developing novel anticancer therapies [95].

## Essential oils and natural volatiles as promising anticancer therapy

Essential oils exhibit significant antioxidant activity due to their rich content of terpenes, terpenoids, and phenylpropanoids, which have demonstrated anticancer properties in human tumor cell lines and are produced as a mix of secondary metabolites in aromatic plants to attract pollinators and protect against environmental stressors [96]. Research indicates that low doses of EOs can alter cancer cell membrane permeability and metabolism, leading to increased ROS levels that induce apoptosis [97]. EOs target critical cellular pathways involved in growth and proliferation, modulating factors such as NF-κB and AKT, ultimately enhancing apoptotic processes in tumor cells [6]. The literature supports the role of EOs in improving cancer treatment efficacy through their multifaceted mechanisms of action, making them promising candidates for further therapeutic development.

**Fig. 6:**
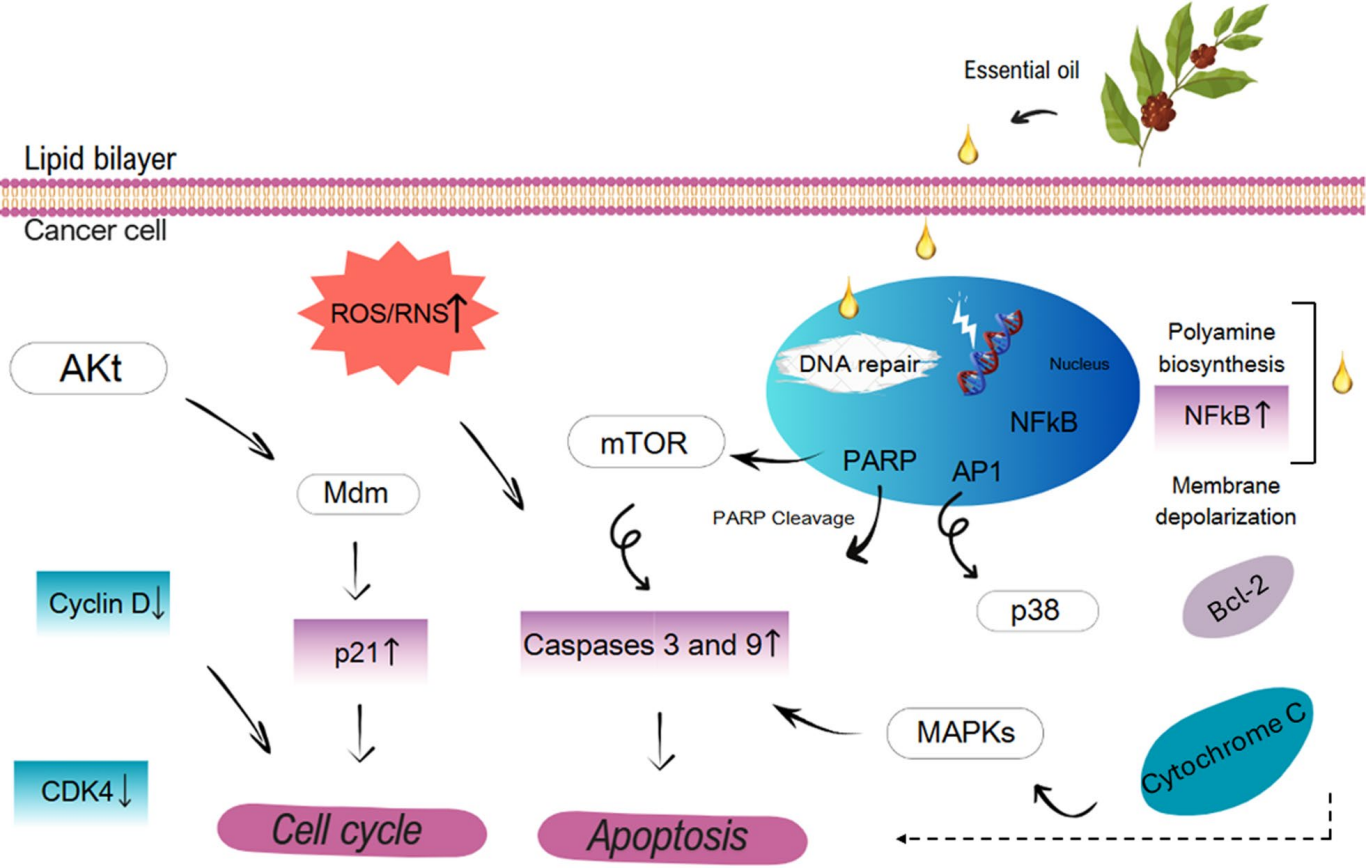
Cellular targets involved in anticancer activity of essential oils. Adapted from Gautam et al. [6]

Figure 6 shows cellular targets involved in cell growth, proliferation, and metabolic pathways of EOs. In brief, (1) minimal doses of EOs alter membrane permeability and crosses the cell membrane, (2) increased levels of ROS and reactive nitrogen species (RNS) in cancer cells lead apoptosis, (3) inhibit activities of AKT, mTOR, and MAPK pathways at different steps by EOs leads to corresponding up-/downregulation of various important biomolecules, (4) Altered expression of NF-κB and its further binding to DNA result in apoptosis in cancer cells, (5) dephosphorylation of AKT by the action of EOs results both overexpression of p21 and cell cycle arrest, which induces apoptosis by increasing caspases level or results in binding to cyclins, respectively (6) mitochondrial stress induced by EOs leads both activation of Bcl-2 and membrane depolarisation, these results in enhanced release of cytochrome-C to the cytoplasm, which induces apoptotic cell death in tumour cells, and finally, (7) EOs modulate DNA repair mechanisms due to that they act as DNA polymerase inhibitors and lead to poly (ADP-ribose) polymerases (PARP) cleavage, which promote cell death by apoptosis in cancer cells.

Currently, EOs from various plant species are recognized as a rich source of potential anticancer agents, particularly from the families Annonaceae, Asteraceae, Lamiaceae, Lauraceae, Myrtaceae, Pinaceae, Poaceae, Rutaceae, and Zingiberaceae. Researchers worldwide have documented the biological effects of these oils, and their anticancer activities summarized in Table 1. The table categorizes the species according to their respective botanical families and presents data on their effects against various cancer cell lines. Among the most commonly evaluated cancer types are breast cancer (including MCF-7, T47D, MCF-10A, MDA-MB-231, SKBR3, BT474), cervical cancer (HeLa, SiHa), colorectal cancer (HCT-116, HT-29, SW48, SW480, 502713, Caco2), gastric cancer (AGS, MGC-803), glioblastoma (M059J, SF763), leukemia (HL-60, K562, Jurkat, THP-1), liver cancer (HepG2, J5), lung cancer (NCI-H358M, A549), nasopharyngeal cancer (KB), neuroblastoma (IMR-32), ovarian cancer (OVCAR-8), oral cancer (OEC-M1, YD-8), prostate cancer (PC3, PC-3M, LNCap), pancreatic cancer (MIA PaCa-2, Panc-28, BxPC3, DANG), renal cancer (ACHN), and skin cancer (A375, A431, MV3, C32, UACC-62). This overview highlights the diverse anticancer potential of EOs across multiple tumor types.

**Table 1:** Anticancer properties of essential oils obtained from plant species

Family	Scientific name	Anticancer effects	Tested model	References
Annonaceae	*Cardiopetalum calophyllum* Schltdl	Antiproliferative	MCF-7, HeLa, and M059J cell lines	[98]
	*Monodora myristica* (Gaertn.) Dunal	Cytotoxic	MCF-7 cell line	[99]
	*Xylopia aethiopica* (Dunal) A. Rich	Cytotoxic	MCF-7 cell line	[99]
	*Xylopia frutescens* Aubl	Cytotoxic, antitumor	OVCAR-8, NCI-H358M), and PC-3M cell lines Antitumor activity evaluated in mice implanted with Sarcoma 180 (S180) tumor cells	[100]
	*Xylopia parviflora* (A. Rich) Benth.)	Cytotoxic	MCF-7 cell line	[99]
Amaryllidaceae	*Allium sativum* L	Production of intracellular ROS, apoptotic inducer, and potentiation differentiation	HL-60 cell line	[101]
Apiaceae	*Anethum graveolens* L	Apoptotic inducer	A549 cell line	[102]
	*Artemisia iwayomogi* Kitam	Apoptotic inducer (MAPKs mediate)	KB cell line	[103]
Asteraceae	*Helichrysum italicum* (Roth) G.Don	Cytotoxic	A375 cell line	[104]
	*Santolina chamaecyparissus* L	Cytotoxic	MCF-7 cell line	[105]
	*Tagetes minuta* L	Cytotoxic	KB and HepG2 cell lines	[106]
	Tanacetum sinaicum* (Fresen.) Delile ex K.Bremer & Humphries*	Antiproliferative, antimetastatic	HeLa and MCF7 cell lines	[107]
	*Tarchonanthus* *camphoratus* L	Antiproliferative. Cell cycle arrest at the G1/S phase, and apoptosis	MCF-7, HepG2, and A549 cell lines	[108]
Araliaceae	*Schefflera heptaphylla* (L.) Frodin	Antiproliferative	MFC-7, HepG2, and A375 cell lines	[109]
Burseraceae	*Boswellia sacra* Flueck	Cytotoxic Antiproliferative and pro-apoptotic against pancreatic tumors in a mouse model	MIA PaCa-2, Panc-28, BxPC3, and DANG cell lines Heterotopic xenograft mouse model	[110]
	*Commiphora guidottii* Chiov. ex Guid	Cytotoxic	MCF-10A, MCF-7, MDA-MB-231, SKBR3, and BT474 cell lines	[111]
	*Protium ovatum* Engl	Antiproliferative	MCF-7, HeLa, and M059J cell lines	[98]
Cupressaceae	*Calocedrus formosana* Kurz	ROS-mediated autophagy, apoptosis	HCT116 p53-wt and HCT116 p53-null cell lines	[112]
	*Juniperus communis* L	Cytotoxic	HepG2, MCF-7, A549, SiHa, and A431 cell lines	[113, 114]
Cyperaceae	*Cyperus rotundus* L	Cytotoxic	HeLa, MCF-7, HCT-116, and HepG2 cell lines	[115–117]
Illiciaceae	*Illicium verum* Hook.f	Antiproliferative, Cytotoxic, apoptosis initiation	MDA-MB-231 cell line	[118]
Lamiaceae	*Cunila angustifolia* Benth	Cytotoxic	MCF-7 cell line	[119]
	*Lavandula stoechas* L	Anti-tumour	AGS, MV3, and MDA-MB-231 cell lines	[120]
	*Melissa officinalis* L	Cytotoxic	K562 and B16F10 cell lines	[121]
	*Nepeta curviflora* Boiss	Antiproliferative and anti-migratory efficacy	HeLa cell line	[36]
	*Ocimum basilicum* L	Cytotoxic	KB and HepG2 cell lines	[106]
	*Ocimum sanctum* L	Antiproliferative	MCF-7 cell line	[122]
	*Ocimum tenuiflorum* L	Antiproliferative, inhibition of cell migration and invasion	AGS cell line	[123]
	*Origanum mejorana* L	Protector autophagy and apoptotic cells death via the activation of the p38 MAPK signaling pathway	HT-29 cell line	[124]
	*Origanum minutiflorum* O. Schwarz & P. H. Davis	Antiproliferative	MCF-7, A-549, and HepG2 cell lines	[125]
	*Origanum vulgare* L	Antiproliferative	HepG2, MDA-MB-231, and BC cell lines	[126, 127]
	*Rosmarinus officinalis* Spenn	Antioxidant, antiangiogenic, epigenetic actions, regulation of the immune response and anti-inflammatory response. Modification of specific metabolic pathways, and increased expression of onco-suppressor genes	HeLa and MCF-7 cell lines	[59, 128]
	*Thymus vulgaris* L	Antiproliferative	MCF-7, PC3, HCT-116, and A549 cell lines	[113]
	*Thymus algeriensis* Boiss. & Reut	Apoptotic inducer	HCT116 cell line	[129]
Lauraceae	*Cinnamomum zeylanicum* J. Presl	Cytotoxic, proapoptotic	K562, MCF-7, and MDA-MB-231 cell lines	[130, 131]
	*Laurus nobilis* L	Cytotoxic	C32 and ACHN cell lines	[132]
	*Litsea cubeba* (Lour.) Pers	Antiproliferative	K562 and MDA-MB-231 cell lines	[130]
	*Machilus mushaensis* (Lu) Y. C. Liu	Antiproliferative	OEC-M1, J5, A549, HT-29, UACC-62, and K562 cell lines	[133]
	*Mentha spicata* L	Antiproliferative	T47D, MCF-7, and HCT-116 cell lines	[134]
	*Phoebe bournei* (Hemsl.) yang	Antiproliferative	HL-60, MCF-7, and SW480 cell lines	[135]
Myrtaceae	*Campomanesia adamantium* O. Berg	Antiproliferative	MCF-7, HeLa, and M059J cell lines	[98]
	*Eucalyptus benthamii* Maiden & Cambage	Antiproliferative	Jurkat cell line	[136]
	*Eucalyptus globulus* Labill	Antiproliferative	SW48 cell line	[137]
	*Melaleuca alternifolia* (Maiden & Betche) Cheel	Apoptotic inducer, increased mitochondrial superoxide production, loss of mitochondrial membrane potential, caspase 3/7 activation, cell cycle arrest in the G0/1–phase, autophagy	LNCaP and MCF-7 cell lines	[138]
	*Psidium guajava* L	Antiproliferative	KB cell line	[139]
	*Syzygium aromaticum* (L.) Merr. & L.M.Perry	Antiproliferative	PC3, HCT-116, A549, and MCF-7 cell lines	[113, 140]
Pinaceae	*Abies pindrow* (Royle ex D.Don) Royle	Antiproliferative	T47D, MCF-7, and A549 cell lines	[141]
	*Cedrus atlantica* (Endl.) Manetti ex Carriere	Antiproliferative	K562 cell line	[142]
	*Cedrus deodora* (Lamb.) G.Don	Antiproliferative	K562 cell line	[142]
	*Cedrus libani* A.Rich	Antiproliferative	K562 cell line	[142]
	*Pinus densiflora* Siebold & Zucc	Antiproliferative, Apoptotic inducer via ROS generation and activation of caspases	YD-8 cell line	[143]
	*Pinus koraiensis* Siebold & Zucc	Antiproliferative, inhibit migration, apoptosis inducer	MGC-803 cell line	[144, 145]
Poaceae	*Cymbopogon citratus* (DC.) Stapf	Antiproliferative	LNCaP, PC-3, SF-767, and SF-763 cell lines	[146]
	*Cymbopogon flexuosus* (Nees ex Steud.) Watson	Apoptotic inducer	Calu-1, HepG2, 502713, and IMR-32 cell lines. Ehrlich Ascites Carcinoma, and Sarcoma	[96, 147]
	*Cymbopogon giganteus* Chiov	Antiproliferative	LNCaP, PC-3, SF-767, and SF-763 cell lines	[146]
	*Cymbopogon nardus* (L.) Rendle	Antiproliferative	LNCaP cell line	[148]
Rutaceae	*Cedrelopsis grevei* Baill	Cytotoxic	MCF-7 cell line	[149]
	*Zanthoxylium schinifolium* Siebold & Zucc	Antiproliferative, apoptotic inducer	HepG2 cell line	[150]
Theaceae	*Camellia sinensis* (L.) Kuntze	Cytotoxic	HepG2, MCF-7, and HCT-116 cell lines	[151]
Verbenaceae	*Lippia citriodora* (Lam.) Kunth	Antiproliferative	A375, HepG2, MCF-7, Caco2, THP-1	[152]
Zingiberaceae	*Curcuma aeruginosa* Roxb. LC	Cytotoxic, apoptosis inducer, cell migration	K562 and MCF-7 cell lines	[153]
	*Curcuma wenyujin* Y.H.Chen & C.Ling	Antiproliferative, apoptotic inducer	HepG2 cell line	[154]
	*Zingiber officinalis* Roscoe	Cytotoxic	HeLa cell line	[155, 156]
	*Zingiber striolatum* Diels	Cytotoxic	K562, A549, and PC3 cell lines	[157]

Cell lines evaluated: breast cancer (MCF-7, T47D, MCF-10A, MDA-MB-231, SKBR3, BT474, T47D), cervical cancer (HeLa, SiHa), colorectal cancer (HCT-116, HT-29, SW48, SW480, 502713, Caco2), gastric cancer (AGS, MGC-803), glioblastoma (M059J, SF763), leukemia (HL-60, K562, Jurkat, THP-1), liver cancer (HepG2, J5), lung cancer (NCI-H358M, A549), nasopharyngeal cancer (KB), neuroblastoma (IMR-32), ovarian cancer (OVCAR-8), oral cancer (OEC-M1, YD-8), prostate cancer (PC3, PC-3M, LNCap), pancreatic cancer (MIA PaCa-2, Panc-28, BxPC3, DANG), renal cancer (ACHN), skin cancer (A375, A431, MV3, C32, UACC-62), and lymphocytes collected from patients with chronic lymphocytic leukemia (CLL)

**Fig. 7:**
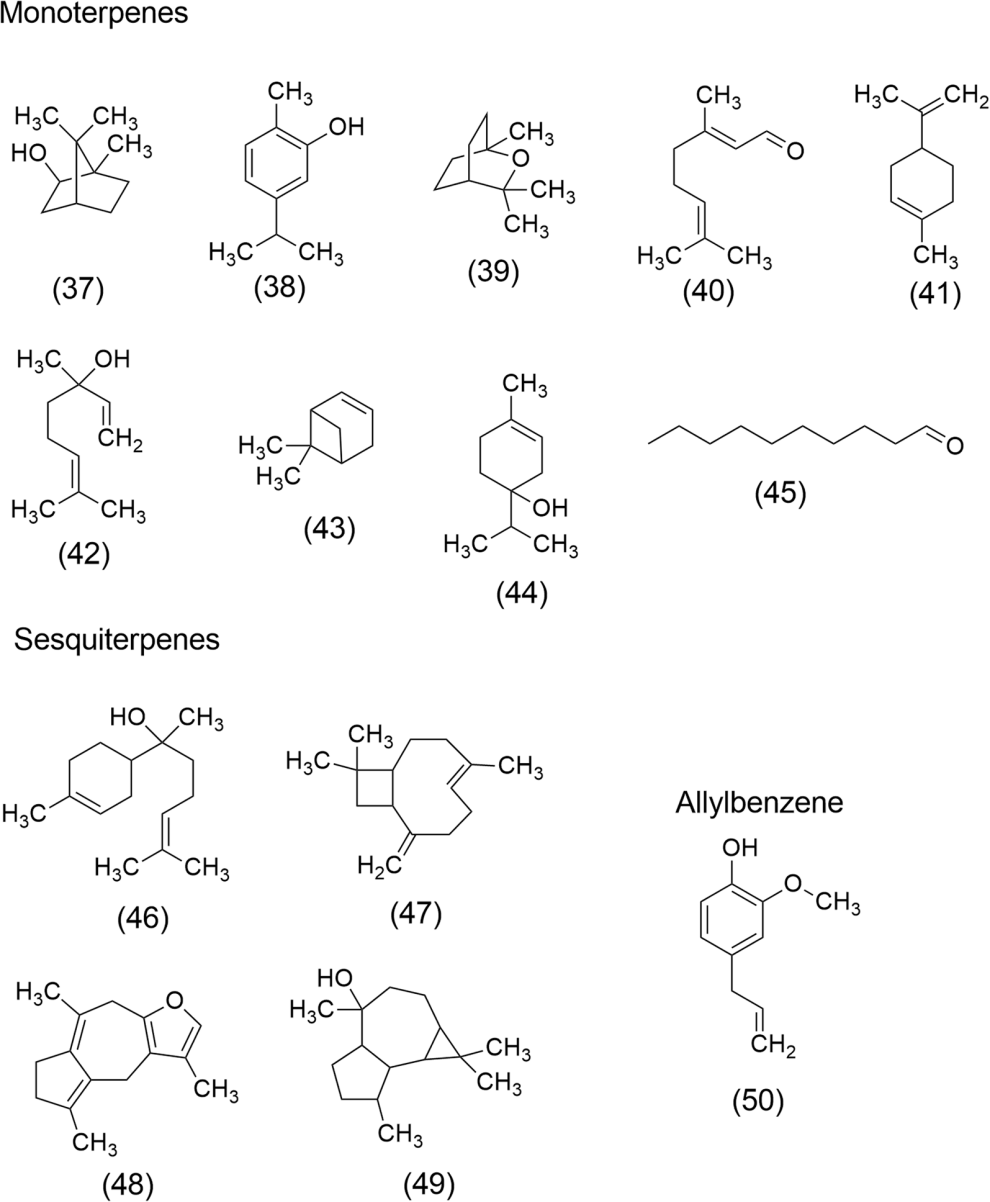
Volatile compounds of EOs with anticancer properties: Borneol (37), Carvacrol (38), 1,8-Cineole (39), Citral (40), Limonene (41), Linalool (42), α-Pinene (43), Terpinen-4-ol (44), Decanal (45), α-Bisabolol (46), β-Caryophyllene (47), Isofuranodiene (48), Viridiflorol (49) and Eugenol (50)

The versatility of EOs has led to the identification of some important volatile compounds with remarkable anticancer activity, including Borneol (37), Carvacrol (38), 1,8-Cineole (39), Citral (40), Limonene (41), Linalool (42), α-Pinene (43), Terpinen-4-ol (44), Decanal (45), α-Bisabolol (46), β-Caryophyllene (47), Isofuranodiene (48), Viridiflorol (49) and Eugenol (50). The chemical structures of these compounds are shown in Fig. 7.

Borneol (37), is a promising monoterpenoid isolated from EOs of many aromatic plants including wormwood (*Artemisia iwayomogi* Kitam*.*, Asteraceae), camphor bush (*Tarchonanthus camphorathus*, Asteraceae), and lemongrass (*Cymbopogon flexuosus* (Nees ex Steud.) Watson., Poaceae), promoting apoptosis in human glioma cells through downregulation of Bcl-2 expression and upregulation of Bax and caspase-3, respectively [108].

Carvacrol (38) is a monoterpene phenol obtained from EOs of an abundant number of plant species, particularly of Lamiaceae and Rutaceae families, including oregano (*Origanum vulgare* L.), thyme (*Thymus vulgaris* L.), and wild bergamot (*Citrus aurantium* L. var. bergamia). Its hydrophobic nature is due to the benzene ring; however, it has been demonstrated that methyl and isopropyl substituents permit the moiety to bind with guanine in DNA [158]. (38) acts as cytotoxic, genotoxic, and proapoptotic in tumor cell lines of the breast, colon, liver, and lung [159].

1,8-Cineole (39) is an achiral volatile component of many plants, including salvia (*Salvia officinalis* L.) and eucalyptus (*Eucalyptus globulus* Labill.). This monoterpene induces proapoptotic effects against A2780 ovarian cancer cells [160] and induces G2/M arrest in A431 epidermoid carcinoma cells by upregulation of the p53 apoptotic signaling pathway [161]. Furthermore, apoptotic effects have been observed in Molt 4B acute lymphoblastic leukemia and HL-60 human leukemia cells [162].

Citral (40) or geranial is a common monoterpenoid of oregano (*Origanum vulgare* L.), aromatic litsea (*Litsea cubeba* (Lour.) Pers.), lemon verbena (*Lippia citriodora* Kunth*.*), and lemon balm (*Melissa officinalis* L.), which has apoptotic effect by activation of protein procaspase-3, acts on leukemic cell, prostate cancer (LNCaP, PC-3), glioblastoma (SF-767, SF-763) [146], hepatocellular carcinoma (HepG2), breast adenocarcinoma, colon adenocarcinoma (Caco2), and leukemic monocytes (THP-1) [152].

Limonene (41) is a terpenoid widely distributed in aromatic plant species including calabash nutmeg (*Monodora myristica* (Gaertn.) Dunal.), wild marigold (*Tagetes minuta* L.), common juniper (*Juniperus communis* L*.*), oregano (*Origanum vulgare* L.), cinnamon (*Cinnamomum zeylanicum* J. Presl.), aromatic litsea (*Litsea cubeba* (Lour.) Pers.), bay laurel (*Laurus nobilis* L.), spearmint (*Mentha spicata* L.), Japanese red pine (*Pinus densiflora* Siebold & Zucc.), and lemongrass (*Cymbopogon* spp.). This monoterpene prevents carcinogen-induced breast cancer at the initiation and the promotion/progression stages and acts as an antiproliferative on prostate cancer (LNCaP), breast cancer (MCF-7), lung cancer (A549) cell lines [99, 138].

Linalool (42), a monoterpene of common aromatic plants such as French lavender (*Lavandula stoechas* L.), cinnamon (*Cinnamomum zeylanicum* J. Presl*.*), bay laurel (*Laurus nobilis* L.), and ginger (*Zingiber striolatum* Diels.), acts as an anticancer agent by its cytotoxic and apoptotic properties on amelanotic melanoma and renal cell adenocarcinoma [108, 132], as well as in lung cancer (A549 cell line) [19].

*α*-Pinene (43), which is an organic compound of the polyphenolic group monoterpene (bicyclic monoterpene), is obtained from EOs from mint (*Mentha piperita* L. and *M. arvensis* L.), holy basil (*Ocimum sanctum* L.), and guava (*Psidium guajava* L.). (43) exerts an antiproliferative effect in A549 human lung cancer cells [19], it enhances the activity of natural killer cells via ERK/AKT Pathway [163], exerts cytotoxic effects in PA-1 human ovarian teratocarcinoma cells, and suppress the cell sequence progression along with the programmed cell death [164].

Terpinen-4-ol (44) is a monoterpenoid from tea tree (*Melaleuca alternifolia* (Maiden & Betche) Cheel). (44) showed antiproliferative effects on prostate cancer (LNCaP), and breast cancer (MCF-7) [138]. Shapira et al. [165] demonstrated that this volatile constituent inhibits the growth of cancer cell lines in a dose-dependent manner on colorectal, pancreatic, prostate, and gastric cancer cells.

Decanal (45), is a natural fragrant of the aldehyde group which acts by its antiproliferative properties on lung cancer (A549 cell line) [19].

α-Bisabolol (46), sesquiterpene alcohol, is a volatile compound obtained from lemongrass (*Cymbopogon flexuosus* (Nees ex Steud.) Watson) and chamomile tea (*Matricaria recutita* L. and *M. chamomilla* L.). (46) exerts selective anticancer activity on A549 adenocarcinoma human alveolar basal epithelial cells by cycle arrest, mitochondrial apoptosis, and inhibition of PI3K/AKT signaling pathways [39]. Further, (46) inhibits mammary tumors (4T1cells) in vitro and transplanted [147, 166].

β-Caryophyllene (47) is a bicyclic sesquiterpene common in the EOs of *Cannabis sativa* L., Cannabaceae family, (Marijuana); it exerts antiproliferative effects on ACHN and C32 cell lines [132]. (47) alter important pathways for cancer development, including mitogen-activated protein kinase (MAPK), PI3K/AKT/mTOR/S6K1, and STAT3 pathways [167].

Isofuranodiene (48) is a volatile constituent of wild celery or Alexander’s celery (*Smyrnium olusatrum* L., Apiaceae). (48) has been shown to induce apoptosis in colon cancer cells (HCT116) in a time- and concentration-dependent manner, indicating its potential as a model for developing chemopreventive agents [168].

Viridiflorol (49), a sesquiterpenoid obtained from the EOs of *Cardiopetalum calophyllum* Schltdl. (Annonaceae) has shown cytotoxic and apoptotic effects on breast, lung, and brain cancer cell lines [169].

Eugenol (50) is an allylbenzene derived from clove (*Syzygium aromaticum* L., Myrtaceae), a species recognized for its diverse applications, including its use as a food preservative, antioxidant, anti-inflammatory, antimicrobial, and anticancer agent. Eugenol exerts anticancer effects on the lung, colon, gastric, cervical, breast, and melanoma cells through several mechanisms, including apoptosis, cell cycle arrest, and inhibiting migration, metastasis, and angiogenesis in various tumor cells. Additionally, it is widely used as an adjunct treatment for cancer patients undergoing conventional therapies [170].

## Anticancer therapy and essential oils: enhancing efficacy and reducing toxicity

In recent years, EOs have been used to enhance the sensitivity of cancer cells [171], reduce the toxicity associated with anticancer drugs, improve the efficacy of radiotherapy [172], and increase cytotoxic effects when combined with herbal treatments [173]. For example, EO from *Zataria multiflora* Boiss. used in conjunction with doxorubicin, has been shown to enhance the sensitivity of PC3 prostate cancer cells to ROS generation and apoptosis, positioning it as a promising candidate for combinatorial therapy [171]. Furthermore, a nanoemulsion of ginger and frankincense essential oils combined with mitomycin C has enhanced efficacy against malignant cells while reducing individual drug toxicity [174].

Furthermore, encapsulation of green tea EO into nanocarriers has shown the potential to improve therapeutic specificity and efficacy while minimizing toxicity to normal cells [151]. Also, EOs are used as supportive therapies to manage side effects associated with radiotherapy or chemotherapy, such as insomnia and nausea [18]. Finally, combinations of herbal treatments with anticancer properties have shown significant cytotoxic effects on A549 cells compared to cisplatin [173].

These findings represent only a part of recent research in the literature and underline the importance of investigating EOs from aromatic plants as accessible therapeutic strategies for cancer treatment.

## Conclusions

Medicinal and Aromatic plants exert a pivotal role in the development of new alternatives for the treatment of cancer. Extracts, essential oils, or secondary metabolites obtained from plant species have made it possible to elucidate some of the mechanisms involved in cancer chemoprevention or chemotherapy. Essential oils and extracts have been identified with health-enhancing properties due to the high antioxidant capacity of their phytoconstituents, as well as to the unique properties of essential oils, making their ingredients improve the well-being of cancer patients due to increasing the sensitivity of malignant cells to other therapeutic strategies and reducing the adverse effects generated during radio- or chemotherapy.

Future research should prioritize the elucidation of specific molecular pathways through which bioactive phytochemicals exert their anticancer effects. Investigation of their interactions with cellular targets, including enzymes, receptors, and signaling proteins, is necessary to understand how these compounds induce apoptosis, inhibit proliferation, or modulate the immune response. The development of advanced techniques such as proteomics, metabolomics, and bioinformatics is essential to map these interactions and identify potential biomarkers of efficacy and safety.

Further progress in the identification and isolation of specific bioactive phytochemicals is critical for the development of targeted therapies that minimize side effects and maximize efficacy. The development of targeted delivery systems, such as nanoparticles or liposomes, could further improve the therapeutic index of these compounds by enhancing their accumulation in tumor tissues. As the safety and efficacy of plant-derived compounds are established, their integration into standard chemotherapy regimens is likely to expand.

Finally, exploring undervalued plant species may reveal novel anticancer agents, while sustainable sourcing practices will ensure the ecological integrity and long-term availability of these valuable resources in bioprospecting efforts.

## Data Availability

The data presented in this study are available in the manuscript.

## Abbreviations

AKT: Protein kinase B
AMPK: AMP-activated protein kinase
AP-1: Activator protein-1
ATPase: The enzyme that hydrolyses adenosine triphosphate
BMP9: Bone morphogenetic protein 9
COX-2: Cyclooxygenase 2
DLA: Dalton´s Lymphoma Ascites
DNA: Deoxyribonucleic acid
EGF: Epidermal growth factor
EGFR: Epidermal growth factor receptor
EGR-1: Early growth response factor-1
ER+: Estrogen receptor-positive breast cancer
Erα: Estrogen receptor alpha
ERK: Extracellular signal-regulated kinases
EOs: Essential oils
EU: European Union
FAK: Focal adhesion kinase
Fas-R: Fas receptor
GCO: Global Cancer Observatory
GRAS: Generally Recognized as Safe
GST: Glutathione S-transferase
HEC-1A: Human endometrial adenocarcinoma
HDACs: Histone deacetylases
HGF: Hepatocyte growth factor
HIF 1α: Hypoxia-inducible factor 1-alpha
IARC: International Agency for Research on Cancer
IL: Interleukin
HBV: Hepatitis B virus
HCV: Hepatitis C virus
HDACs: Histone deacetylases
HER-2: Human epidermal growth factor receptor-2
HPV: Human papillomavirus
HS-683: Human glioma cell line
LAC: Latin America and the Caribbean
MAP-K: Mitogen-activated protein kinase
MIP: Macrophage Inflammatory Proteins
MKP-3: Mitogen-activated protein kinase phosphatase-3
MNU: *N*-Methyl-*N*-nitrosourea
MMPs: Matrix metalloproteinases
mTOR: Mechanistic target of rapamycin
MTT: Methyl thiazolyl tetrazolium
NFκB: Nuclear factor kappa B
p53WT: Wild type of p53
p70S6K: P70S6 kinase
PAHO: Pan American Health Organization
PAK: P21-activated kinase
PARP: Poly (ADP-ribose) polymerases
PDGF: Platelet-derived growth factor
PI3K: Phosphatidylinositol 3-kinase
PKA: Protein kinase A
PKB: Protein kinase B
pRB: Retinoblastoma protein
PTK: Protein tyrosine kinase
RNS: Reactive nitrogen species
ROS: Reactive oxygen species
TNBC: Triple-negative breast cancer
TNF-α: Tumor necrosis factor alpha
Topo I: Topoisomerase I
Topo II: Topoisomerase II
STAT-3: Signal transducer and activator of transcription-3
US FDA: United State Food and Drug Administration
VEGFR: Vascular endothelial growth factor receptor
WHO: World Health Organization
